# One Year Duration of Immune Response Following a 3rd Booster Dose of mRNA Vaccine Against COVID‐19 in 292 Patients With Hematological Malignancies in University Hospital Ostrava, Czech Republic

**DOI:** 10.1002/cam4.70503

**Published:** 2024-12-22

**Authors:** Ondrej Šušol, Barbora Šušolová, Ondřej Klempíř, Milan Navrátil, Jaromír Gumulec, Zdeněk Kořístek, Juraj Ďuraš, Michal Kaščák, Jana Mihályová, Lukáš Stejskal, Tomáš Jelínek, Petra Richterová, Lenka Szeligová, Hana Plonková, Jana Zuchnická, Barbora Dluhošová, Ivo Demel, David Buffa, Katarína Hradská, Tereza Popková, Ludmila Muroňová, Martin Lachnit, Klára Lančová, Roman Hájek

**Affiliations:** ^1^ Department of Haemato‐Oncology University Hospital Ostrava Ostrava Czech Republic; ^2^ Faculty of Medicine University of Ostrava Ostrava Czech Republic; ^3^ Department of Biomedical Informatics, Faculty of Biomedical Engineering Czech Technical University in Prague Prague Czech Republic; ^4^ Department of Haematology and Blood Transfusion Silesian Hospital in Opava Opava Czech Republic

**Keywords:** Cancer, COVID‐19, Leukemia, Lymphoma, Myeloma, Vaccination

## Abstract

**Aims:**

To evaluate antibody response to mRNA vaccine, identify subgroups with poor response and to determine long‐term antibody durability in hematological patients.

**Materials and Methods:**

We have vaccinated 292 patients with all hematological malignancies with a third dose of mRNA COMIRNATY vaccine with a 12‐month follow‐up period in our center in Ostrava, Czech Republic.

**Results:**

Antibody response for the whole cohort exceeded 74% through the whole 12‐month follow‐up. Lowest seroconversion was observed in CLL cohort (20/41, 48.8%), patients who received anti‐CD20 therapy < 6 months before vaccination (8/30, 26.7%) and BTK inhibitors (3/6, 50.0%). On the contrary, patients with chronic myeloproliferative neoplasms and acute leukemia performed comparably with healthy population (33/33; 100% and 12/13; 92.3%, respectively). We have seen better results if the time interval between anti‐CD20 therapy and additional vaccine dose was longer than 6 months (5/8 patients achieved seroconversion on 4th booster dose after previous failure). Also, 36 patients received a 4th dose of vaccine as a booster with measurable increase in protective antibodies in 50% (18/36).

**Conclusions:**

Additional doses show promise for a well‐timed revaccination even in poor responders. To our knowledge, no study comparable to our work in terms of follow‐up length, vaccine consistency or variety of hematological malignancies and/or treatment has been reported yet. Our findings shed more light on long‐term antibody response to mRNA vaccines against SARS‐CoV‐2 in patients with hematological cancer and bring important data for the evaluation of possible vaccine failure and scheduling of subsequent doses.

## 1 Introduction

Patients with cancer, especially those with hematological malignancies, have an increased risk of contracting COVID‐19 with increased severity and mortality [1]. Since 2020, multiple vaccine types have been made available for public use through emergency authorizations. Since then, numerous studies have sought to prove their efficacy against COVID‐19 in patients with solid or hematological cancer [2, 3, 4, 5, 6, 7]. The mRNA vaccines have proven to be highly efficient with minimal adverse effects [8]. Next, vaccination schemes have been introduced considering novel viral mutations as well as current epidemiological situation, with recommended booster vaccine doses. Some studies have already evaluated the booster effect of additional vaccine doses [9, 10]. However, these studies were usually performed on a narrow cohort of patients with single type of hematological cancer, mostly with a shorter follow‐up period (up to 6 months). We put forward a 1‐year, prospective study on a cohort of 292 patients with all types of hematological malignancies vaccinated with three doses of single type mRNA vaccine in our center. To our knowledge, no study of such range, integrity or follow‐up length has been reported yet.

## 2 Methods

Since 27th September 2022, we have vaccinated all SARS‐CoV‐2 negative patients who have already been vaccinated with two initial doses [11, 12] but have not yet received a 3rd dose of COVID vaccine at that timepoint, without exception. At first, patients with failure to respond to the initial vaccination or who had a rapid loss of protective antibody titers were prioritized. Then, due to a rapidly worsening epidemiological situation in our region, an effort was made to vaccinate all our patients with a RT‐PCR confirmed SARS‐CoV‐2 negativity. In case of RT‐PCR SARS‐CoV‐2 positive patients, vaccination was postponed until at least 7 days without COVID‐19 symptoms confirmed by a negative RT‐PCR test for SARS‐CoV‐2 from nasopharyngeal swab. Antitumor therapy cycles were not postponed for the sake of vaccination, except for patients with acute leukemia, where we preferred vaccinating in‐between treatment cycles due to severe treatment‐related neutropenia [13]. Also, patients with acute leukemia scheduled for allogeneic bone marrow transplant were vaccinated first to diminish the risk for COVID‐19 infection in the peritransplant period, then revaccinated 6 months after the engraftment according to current guidelines [14, 15, 16, 17]. Close relatives of bone marrow recipients were also offered a 3rd dose of vaccine to further lessen the risks of COVID‐19 infection [17]. Patients undergoing high‐dose chemotherapy with autologous stem cell transplant (ASCT) were vaccinated 3 months after the ASCT.

**FIGURE 1:**
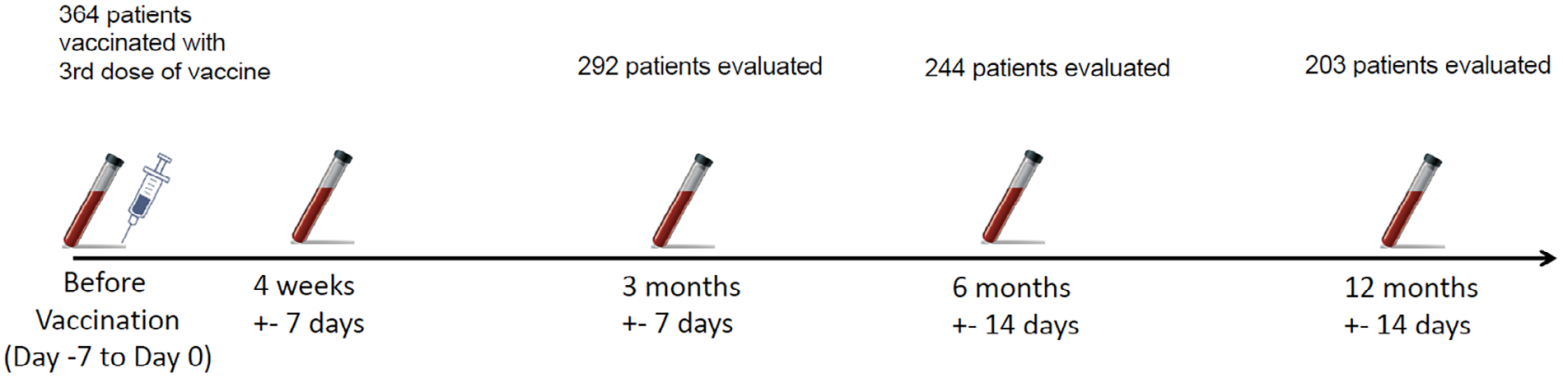
Vaccination and sample collection timeline.

To evaluate the response to third dose of vaccine, blood samples were drawn in our facility at given timepoints (Figure 1) and tested for complete blood count, blood chemistry panel (natrium, potassium, chloride, blood urea nitrogen, creatinine, alkaline phosphatase, aspartate transaminase, alanine aminotransferase, C‐reactive protein, and serum immunoglobulin levels). Patient relevant data (age, sex, diagnosis, treatment status) were extracted from the medical records and correlated with our results.

The blood serum immunoglobulins (IgG, IgM, and IgA) were tested for the detection of anti‐S1/S2 antibodies to SARS‐CoV‐2 using commercial ELISA assays. ELISA assays for the detection of IgG and IgA contained the S1 subunit of the spike protein as antigen and ELISA assay for the detection of IgM contained nucleoprotein (NCP) as antigen (Euroimmun, Lübeck, Germany). A signal‐to‐cut‐off ratio was calculated. Values < 0.9 were regarded as negative, ≥ 0.9 to < 1.1 as borderline and ≥ 1.1 as positive seroconversion. For the detection of neutralizing specific anti‐SARS‐CoV‐2 IgG (BAU) antibodies, we used QuantiVac ELISA IgG kit (Euroimmun, Lübeck, Germany) with the lowest positivity threshold determined at 35.2 BAU/mL. In accordance with our previous work, an in‐house in vivo Virus Neutralization Test (VNT) against the Delta and Omicron variant viruses was performed as described in Šimánek et al. [18]. Baseline VNT positivity was determined as a titer of 20, VNT titer of 80 was determined as strongly positive and titer above 160 was determined as very strongly positive in correlation with data on virus‐specific convalescent donor plasma [19, 20].

A patient's positive response to vaccination was determined as VNT value above the positivity threshold (> 20) regardless of IgG, IgM, or IgA positivity because it was hypothesized that the mechanism of the in vivo neutralization assay best reflects patient's protective antibody activity. For the sake of possible correlation, neutralizing anti‐SARS‐CoV‐2 IgG BAU/mL values were collected (this method became available with the 3rd dose of vaccine). A change from seronegative to seropositive was defined as seroconversion, a booster effect was defined either as a conversion from seronegative to seropositive or two‐fold VNT increase after the third dose [21].

Adverse effects of vaccine were monitored either by telephone controls on Day 7 after vaccination or on next scheduled patient's visit. Patients were asked to report local or systemic adverse effects within 1 week after each dose. For this type of data, no specific adjustment was performed. Patients vaccinated in other caregiving facilities with different types of vaccines against SARS‐CoV‐2 were noted, however data collection and/or evaluation in these patients was not performed.

Additionally, 41 patients have received a 4th dose of Comirnaty BNT 162b2 before the scheduled 12‐month follow‐up sample collection. In these patients, a booster effect of additional dose has been evaluated following the same criteria. Also, 34 patients have received AstraZeneca's Evusheld (formerly AZD7442) before the scheduled 12‐month follow‐up sample collection. These patients were noted for the evaluation of that timepoint.

All presented data analysis was performed using offline scripts developed in the Python 3.8 programming language. We calculated the proportions of patients in each sub‐cohort, conducted detailed quality control inspections of the obtained results, and calculated basic statistical descriptions such as mean, median, interquartile ranges, etc. For the reported correlations, Pearson's correlation coefficient was used. This measures the linear relationship between two continuous variables and ranges from −1.0 to +1.0, with the signs indicating whether the relationship is direct (+) or inverse (−).

## 3 Cohort Description

We have vaccinated all patients regardless of their diagnosis. Our cohort comprises patients with acute leukemia or myelodysplastic syndrome (AL/MDS), multiple myeloma (MM), lymphoma (Hodgkin and non‐Hodgkin), chronic lymphocytic leukemia (CLL) and chronic myeloid neoplasms (cMPN). We have also vaccinated patients with a variety of non‐oncological hematological disorders, but we did not include them in our final evaluation. Vaccination of total 497 patients with initial two doses of COMIRNATY BNT162b2 (Pfizer/BioNTech) took place at our clinic since 1st March 2021 [12]. Since the third dose of vaccine became available in September 2022, we have been able to administer a third booster dose of COMIRNATY BNT162b2 (Pfizer/ BioNTech) to 364 patients. Serological response to the third dose was evaluated in 292 patients. Their characteristics, time from second to third dose along with the rate of missing serology data over time are summarized in Table 1.

**TABLE 1:** Cohort characteristics.

Vaccinated patients	*n* = 362	*x* (%)
Mean time from 2nd to 3rd dose (days)	224 (35–631)	
Evaluated patients	*n* = 292^a^	
Age	18–40 years	10 (3.4)
	40–65 years	128 (43.8)
	≥ 65 years	154 (52.7)
Sex	Male	170 (58.2)
	Female	122 (41.8)
Diagnosis	AL/MDS	25 (8.6)
	CLL	45 (15.4)
	Lymphoma	87 (29.8)
	MG	105 (36)
	cMPN	33 (11.3)
	Active treatment	123 (42.1)
	Evusheld during follow‐up	34 (11.6)
Available data	3 M	292 (100)
	6 M	244 (83.6)
	12 M	203 (69.5)

Abbreviations: 12 M, 12 months post 3rd dose; 3 M, 3 months post 3rd dose; 6 M, 6 months post 3rd dose; AL/MDS, acute leukemia/myelodysplastic syndrome; CLL, chronic lymphocytic leukemia; cMPN, chronic myeloproliferative neoplasms; MG, monoclonal gammopathy.

^a^ Available data for 292/362 vaccinated patients.

Of 25 patients with AL/MDS, 20 (80%) had acute myeloid leukemia (AML) or MDS, 2 (8%) had acute promyelocytic leukemia (APL), and 3 (12%) had acute B‐lymphoblastic leukemia (B‐ALL). Mean age was 56.8 years and 52% (13/25) were male. Patients on ongoing therapy accounted for 60% (15/25), of which 13 patients had AML/MDS, one patient had B‐ALL and one patient had APL. Of 11 patients with AML on ongoing therapy, 2 (18.2%) were treated with venetoclax. Also, we have been able to evaluate 14 patients after allogeneic stem cell transplant (10 for AML, two for B‐ALL and two for MDS).

Of 45 patients with CLL, 71.1% (32/45) were male and mean age was 67.0 years. Ongoing therapy at the time of vaccination had 46.7% (21/45) and 53.3% (24/45) were currently not on treatment. Further characteristics are summarized in Table 2.

**TABLE 2:** Characteristics of CLL and lymphoma patients.

CLL (*n* = 45)	*x* (%)	3 M response (%)	12 M response (%)
Therapy at time of 3rd dose			
Median age (years)	67.0		
Male	32/45 (71.1)		
Female	13/45 (28.9)		
No current treatment	24/45 (53.3)	9/20 (45)	11/17 (64.7)
Active therapy	21/45 (46.7)		
BTKi	6/45 (13.3)	3/6 (50)	3/6 (50)
Venetoclax	7/45 (15.6)	4/7 (57.1)	4/7 (57.1)
Anti‐CD20 treatment	13/45 (28.9)	5/13 (38.5)	6/13 (46.2)
< 6 months before 3rd dose			

Lymphoma (*n* = 87)	*x* (%)	3 M response (%)	12 M response (%)
Median age (years)	59.4		
Male	49/87 (56.3)		
Female	38/87 (43.7)		
Hodgkin's lymphoma	11/87 (12.6)	11/11 (100)	7/11 (63.6)
T Lymphoma	4/87 (4.6)	4/4 (100)	3/3 (100)
Hairy cell leukemia	3/87 (3.4)	3/3 (100)	2/2 (100)
B‐NHL			
DLBCL	18/87 (20.7)	14/18 (77.8)	11/14 (78.6)
FL	29/87 (33.3)	13/29 (44.8)	12/19 (63.2)
MZL	13/87 (14.9)	9/13 (69.2)	6/8 (75)
MCL	6/87 (6.9)	2/6 (33.3)	2/4 (50)
Other*	3/87 (3.4)	1/3 (33)	0/2 (0)
Anti‐CD20 treatment < 6 months before 3rd dose	17/69 (24.6) **	3/17 (17.6)	10/17 (58.8)
Combined response for anti‐CD20 treatment < 6 months before 3rd dose in CLL and lymphoma	*n* = 30	8/30 (26.7)	16/30 (53.3)

Abbreviations: 12 M, twelve months; 3 M, three months; B‐NHL, B‐cell non‐Hodgkin's lymphoma; BTKi, Bruton tyrosine kinase inhibitors; CLL, chronic lymphocytic leukemia; DLBCL, diffuse large B‐cell lymphoma; FL, follicular lymphoma; MCL, mantle cell lymphoma; MZL, marginal zone lymphoma.

* Two patients with Waldenström's macroglobulinemia (WM) and one patient with SLL. The patient with SLL responded at the 3 M timepoint but was then lost to follow‐up. The two patients with WM did not respond.

** Cohort of 69 patients with B‐NHL, WM and SLL.

Of 87 patients with lymphoma, 56.3% (49/87) were male and mean age was 59.4 years. Most patients (79.3%, 69/87) had non‐Hodgkin's B‐lymphoma (B‐NHL), of which 26% (18/69) had DLBCL and 73.9% (51/72) had indolent B‐cell lymphoma. Also, 17 patients (24.6%) with B‐NHL received anti‐CD20 treatment less than 6 months prior to the 3rd dose. Further cohort characteristics are summarized in Table 2.

Of 33 patients with cMPN, 39.4% (13/33) had chronic myeloid leukemia (CML), 30.3% (10/33) had polycythemia vera (PV), 18.2% (6/33) had primary myelofibrosis (PMF), 3 (9.1%) had essential thrombocythemia (ET) and one patient had myelodysplastic/myeloproliferative neoplasm (MDS/MPN). Mean age was 62.3 years and 51.5% (17/33) were male. Twenty patients (60.7%) were on active treatment at the time of vaccination (11 on BTKi and nine on symptomatic therapy).

Of 105 patients with monoclonal gammopathy, 93.3% (98/105) had multiple myeloma, 3.8% (4/105) had MGUS, two patients (1.9%) had AL amyloidosis, and one patient had plasma cell leukemia. Mean age was 67.0 years and 57.1% (60/105) were male. At the time of vaccination 75.2% (79/105) patients were on ongoing therapy and 19 patients (18.1%) received anti‐CD38 therapy less than 6 months prior to vaccination. Also, 73 patients (69.5%) had immune paresis in at least one of the immunoglobulin classes (IgM, IgG, or IgA).

In 36 patients who had received a 4th dose of Comirnaty BNT 162b2 before the scheduled 12‐month follow‐up, sample collection and evaluation for booster effect was performed (Table 3).

**TABLE 3:** patients who received Evusheld and 4th dose of vaccine.

EVUSHELD before the end of follow‐up (*n* = 34)	
MM	22
Lymphoma	7
CLL	3
cMPN	1
AL/MDS	1
4th DOSE (*n* = 36)	
MM	12
Lymphoma	12
cMPN	5
CLL	6
AL/MDS	1
Booster effect	
Anti‐CD20	5/8 (62.5%)
Anti‐CD38	1/4 (25%)

Abbreviations: AL/MDS, acute leukemia/myelodysplastic syndrome; Anti‐CD20, patients treated with anti‐CD20 therapy < 6 months prior to 3rd dose; Anti‐CD38, patients treated with anti‐CD38 therapy < 6 months prior to 3rd dose; CLL, chronic lymphocytic leukemia; cMPN, chronic myeloproliferative neoplasms; MG, monoclonal gammopathy.

## 4 Results

Overall response in the whole cohort was 75.2% (218/290), 74.2% (181/244), and 78.1% (157/201) for respective timepoints (Table 4, Figure 4).

**TABLE 4:** VNT positivity at different timepoints.

	Baseline	3 M	6 M	12 M
AL/MDS	9/22 (40.9%)	20/21 (95.2%)	15/18 (83.3%)	12/13 (92.3%)
CLL	4/45 (8.9%)	20/41 (48.8%)	22/43 (51.2%)	23/42 (54.8%)
Lymphoma	25/81 (30.9%)	57/87 (65.5%)	46/71 (64.8%)	43/60 (71.7%)
MG	39/99 (39.4%)	87/105 (82.9%)	70/84 (83.3%)	59/64 (92.2%)
cMPN	13/28 (46.4%)	33/33 (100%)	28/28 (100%)	20/23 (87%)
Total	114/274 (41.6%)	218/290 (75.2%)	181/244 (74.2%)	157/201 (78.1%)

Abbreviations: AL/MDS, acute leukemia/myelodysplastic syndrome; CLL, chronic lymphocytic leukemia; cMPN, chronic myeloproliferative neoplasms; MG, monoclonal gammopathy; VNT, virus neutralization test.

In AL/MDS cohort, 40.9% (9/22) had a positive baseline VNT with median 198.5 BAU/mL; data from three patients could not be obtained. The overall response after the 3rd dose was 88.0% (22/25) with median 940.9 BAU/mL (0.01–989.1). In long‐term follow‐up at 12 months after vaccination, this response remained as high as 92.3% (12/13) with median 1000.5 BAU/mL (691.0–1224.0). Both two patients treated with venetoclax responded (VNT 2560 and 640; 971.5 and 889.1 BAU/mL, respectively). The response in 14 patients after allogeneic stem cell transplant was 100% for all specific timepoints with an average of 851.2 BAU/mL. One patient with suboptimal response received Evusheld with a measurable increase in nAb levels.

Of 45 CLL patients, 8.9% (4/45) patients had a positive baseline VNT with median 126.9 BAU/mL at the time of vaccination. These values increased to 48.8% (20/41) with median 689.97 BAU/mL (0.01–1038.0) 3 months after the 3rd dose of vaccine, expressing a seroconversion rate of 39% (16/41). We have seen an increase to 56.1% (23/41) with median 760.61 BAU/mL (0.01–1066.0) at the 12‐month follow‐up timepoint. In a sub‐cohort, 13 patients treated with anti‐CD20 < 6 months prior to vaccination, 6 patients treated with Bruton's tyrosine kinase inhibitor (BTKi), and 7 patients treated with venetoclax were evaluated (see Table 2). Response rates for anti‐CD20, BTKi and venetoclax were 38.5% (5/13; 214.8 BAU/mL), 50% (3/6; 467.3 BAU/mL), and 57.1% (4/7; 558.6 BAU/mL), respectively. In the 12‐month follow‐up, response rates remained the same except for the anti‐CD20 cohort, where we saw an increase to 46.2% (6/13). A multivariate logistic regression model for different therapies, sex and age was performed to determine negative predictors of vaccination response. A negative predictive value was only determined for anti‐CD20 therapy (*p* = 0.35) in CLL cohort.

Of 87 patients with lymphoma, 40.7% (33/81) had baseline positive VNT with median 510.3 BAU/ml; data from six patients could not be obtained. Response rate after the third dose was 65.5% (57/87) with median 917.4 BAU/mL (0.0–1041.0) and 73.3% (44/60) with median 846.2 BAU/mL (23.7–1134.6) at 12 months. Results for different subgroups are summarized in Table 2. In 17 patients with previous anti‐CD20 treatment, 3 (17.6%) had baseline VNT positivity before 3rd dose of vaccine. This increased to 4 (23.5%) at the 3‐month follow‐up timepoint. At 12‐month follow‐up, the overall response for this sub‐cohort was 58.8% (10/17).

Combined response in 30 patients with lymphoma or CLL, treated with anti‐CD20 < 6 months before vaccination was 26.7% (8/30) and 53.3% (16/30) on given timepoints (see also Table 2).

Of 33 patients with cMPN, 21 (63.6%) had baseline positive VNT with median 286.5 BAU/ml. The overall response 3 months after the 3rd dose was 100% and remained 100% at the 6‐month follow‐up timepoint with median 911.5 BAU/mL (96.9–1002.0). We saw a drop to 87% (20/23) with median 955.4 BAU/mL in a 12‐month follow‐up.

Of 105 patients with monoclonal gammopathy, 39.4% (39/99) had baseline positive VNT with median 380.2 BAU/mL; data from six patients could not be obtained. After the third dose, seroconversion rate was 81.9% (86/105) with median 918.3 BAU/mL (21.0–1038.6). At the 12‐month follow‐up, seroconversion rate was 92.2% (59/64) with median 947.0 BAU/mL (0.01–1192.0). In a sub‐cohort of 19 patients treated with anti‐CD38, 18 (94.7%) had a persistent serological response 12 months after vaccination. One patient who did not respond received Evusheld with a measurable increase in neutralizing antibodies afterwards.

Thirty‐six patients have received a 4th dose of Comirnaty BNT 162b2 before the scheduled 12‐month follow‐up sample collection. We saw a booster effect in 50% (18/36) patients; however, all patients retained their seroconversion after the 4th dose. For results in specific therapies (anti‐CD20 and anti‐CD38) see Table 4. Importantly, eight patients with baseline negative protective antibodies mounted a serological response after the fourth dose. These were four patients with lymphoma (three indolent lymphomas and one DLBCL), two patients with multiple myeloma and two patients with CLL.

## 5 Discussion

We have seen encouraging results for the whole cohort exceeding 75% positive seroconversion. In comparison with serological responses 6 months after the initial two doses (61.6%, 116/190) we saw an encouraging increase in positive seroconversion rates throughout the whole cohort. This comparison is based solely on VNT levels because the BAU assessment only became available later. Nevertheless, we consider the VNT a valid method for seroconversion assessment based on other studies that confirmed its potential for the evaluation of strain‐specific neutralizing antibody activity [22]. Moreover, our findings were in accordance with analogical studies [23, 24]. A short‐lived antibody response in immunocompromised patients including patients with hematologic malignancy has been reported [25], but this was only partially true in our cohort, because we have observed significant differences in vaccination response rates, antibody titers and response durability in different patient subgroups.

Another study [23] describes 167 CLL patients vaccinated with up to eight subsequent doses, of which 44 (20.5%) were on ongoing treatment and 14 (6.5%) had received anti‐CD20 therapy in the last 12 months. Of 68 patients with CLL who were seronegative after two doses, 27 (39.7%) achieved positive seroconversion after the 3rd dose with a reported rise in BAU levels after each dose. However, a significant positive seroconversion capable of neutralizing activity (> 5000 AU/mL for the Omicron variant) was only achieved in 35.6% of patients. This is partly in accordance with our data, because we have observed a similar rise in seropositivity after the 3rd dose (39%) but with better long‐term durability (more than 50%) in the 12‐month follow‐up. The BAU/mL levels in our cohort remained at a satisfactory magnitude, even with a long‐term increase. However, due to a different result presentation of our in vivo VNT, we were unable to directly transfer our results to BAU neutralizing activity. To that end, we attempted to evaluate the correlation between the 3rd dose VNT and BAU, but we could not state a clear linear relationship between the VNT/IgG ratio and BAU, both at an overall level (i.e., across all patients) and within individual subgroups. The individual correlation coefficients between pairs of parameters were insignificant, reaching values around rho = 0. A potential solution for achieving effective transformation in our future work could be the use of a nonlinear model to explain these transformations. Nevertheless, given the methodology of our in‐house VNT testing, we assumed a sufficient neutralization activity in seropositive patients regardless of BAU/ml quantification.

An evaluation of 20 CLL patients treated with BTKi or anti‐CD20 treatment reported a positive seroconversion in 6/9 (66.7%) and 6/14 (42.9%) patients, respectively [24]. Similar results were achieved in the MARCH study [26] with a 37.5% response in eight patients on BTKi treatment, 50.0% response in 4 patients on venetoclax and 55.0% response in patients treated with anti‐CD20 < 6 months prior to vaccination (only 21.7% had BAU threshold above the bottom 10% measurable antibody level). Worse results, but better positive seroconversion for longer period between anti‐CD20 and vaccination were reported by Mehta‐Shah et al. [10]. These results match our own; importantly, in accordance with the MARCH study, we have observed lower VNT and BAU levels and a shorter time to losing seropositivity after the third dose in CLL patients. Accordingly, we have observed lower VNT and BAU levels and a shorter time to losing seropositivity after the 3rd dose in CLL patients compared to other subgroups, which is in accordance with other reports [26]. This is of importance, more so because recent robust data from a large, multicenter prospective Spanish study [27] have put forth an overall higher cumulative risk of breakthrough SARS‐CoV‐2 infections in CLL patients.

Unlike other studies [23], we have identified only one potential negative predictor in a multivariate analysis for vaccination outcome in CLL patients, which was anti‐CD20 treatment < 6 months before vaccination. However, for CLL and B‐NHL combined, a strong negative predictor was identified for anti‐CD20 treatment (*p* < 0.05) and CLL (*p* = 0.017), which was in accordance with previously published data [24].

Among lymphoma patients there was an expected difference between HL patients and NHL patients. Whereas HL patients performed well with near‐normal seroconversion rates, NHL patients have shown less encouraging results compared to the rest of our study cohort (with the exception of CLL). Most importantly, the B‐NHL cohort counted among the frailest in terms of serological response to vaccination, even with the administration of booster doses with suboptimal response rates. Low positive seroconversion rates for B‐NHL patients have already been reported for initial two doses, especially low in patients who had a short‐term history (less than 6 months) of receiving anti‐CD20 treatment before vaccination [3, 28, 29, 30]. Our findings support the results already published by other authors—low positive seroconversion rate with lesser antibody titers and a short‐lived anti‐SARS‐CoV‐2 antibody persistence along with significantly suppressed positive seroconversion after anti‐CD20 treatment after the third booster dose [10, 31, 32]. We have seen an improvement in positive seroconversion rates in the 12‐month follow‐up, especially in patients previously treated with anti‐CD20 treatment. This was probably due to a longer time interval from anti‐CD20 treatment. This was demonstrated in five of eight patients treated with anti‐CD20 treatment < 6 months prior to third dose, who have mounted an antibody response following the fourth booster dose. Even though a small cohort, our results show more promise than results by other authors [10].

Long‐term serological kinetics in 103 patients with multiple myeloma 6 months after having received a third dose of vaccine have been reported [33]. At 6 months, the serological responses remained as high as 99% with a 10‐fold increase in antibody levels after the third dose. Our evaluation showed less promising results (81.9%) with an increase to 92.2% in the 12‐month follow‐up. However, our results are in accordance with a comparable study [34] which reports an overall response of 83.6% with an average BAU 370.4 U/mL. Despite similar overall response, we have observed higher BAU/mL values (median 918.3) in our cohort. Contrary to our expectations, we saw very encouraging results comparable with healthy population in 19 patients vaccinated after preceding anti‐CD38 treatment. In our observation, anti‐CD38 treatment did not prove to be an inhibiting factor for vaccine efficacy.

The positive seroconversion rates seen in cMPN and AL/MDS groups in our study were comparable to healthy population [35, 36, 37], matching previously published results [38, 39]. In the 12‐month follow‐up, we have seen an increase in antibody levels and high protective antibody persistence (87% and 92.3%, respectively) with 955.4 and 1000.5 BAU/mL, respectively. These results are slightly worse, but still comparable to healthy population and confirm good protective antibody response and its long‐term persistence in patients with myeloid malignancies.

Even though the strict booster effect criteria after the additional fourth dose have only been met in 43.9% patients, we have seen an encouraging persistency with unwavering IgG, BAU, and VNT values suggesting a prolongation in positive serological response rather than booster effect, with an antigen immune update covering current pathogenic variants of SARS‐CoV‐2.

## 6 Conclusion

Our data confirm an impaired response to mRNA vaccines in hematological patients, putting forth most vulnerable groups to vaccine failure (CLL, B‐NHL, anti‐CD20, and BTKi treatment). Seasonal revaccination with a booster dose with respect to current viral strain prevalence should remain the cornerstone of prophylaxis. Although some authors suggest revaccination every 4 weeks until a seroconversion is achieved [40], such an approach can be quite inconvenient for patients and caregivers alike. Based on our data and recommendations by other authors [41], we suggest an antibody level confirmation 4 weeks after each booster dose in patients at risk and non‐responders with the administration of depot monoclonal antibodies in patients who fail to mount a sufficient response. Nevertheless, further data will be needed to establish an efficient, most likely patient‐tailored vaccination policy.

## Author Contributions

**FIGURE 2:**
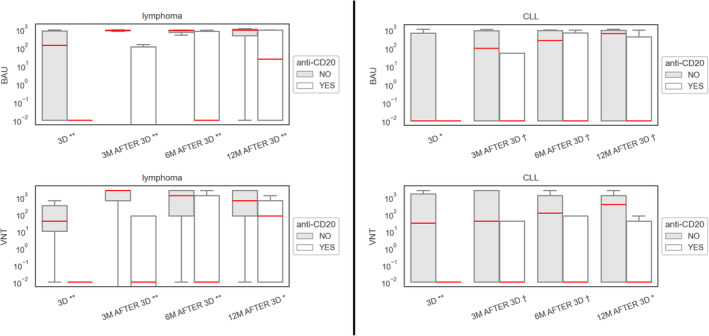
Response in lymphoma and CLL on anti‐CD20 therapy. *p* < 0.05; **p < 0.01; † *p* value nonsignificant. We used the Mann–Whitney *U* test to assess a statistically significant difference in the VNT/BAU values.

**FIGURE 3:**
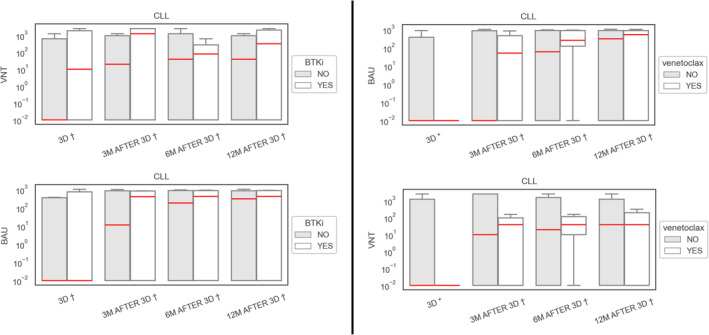
Response in CLL on BTKi and venetoclax. *p* < 0.05; **p < 0.01; † *p* value nonsignificant. We used the Mann–Whitney *U* test to assess a statistically significant difference in the VNT/BAU values.

**FIGURE 4:**
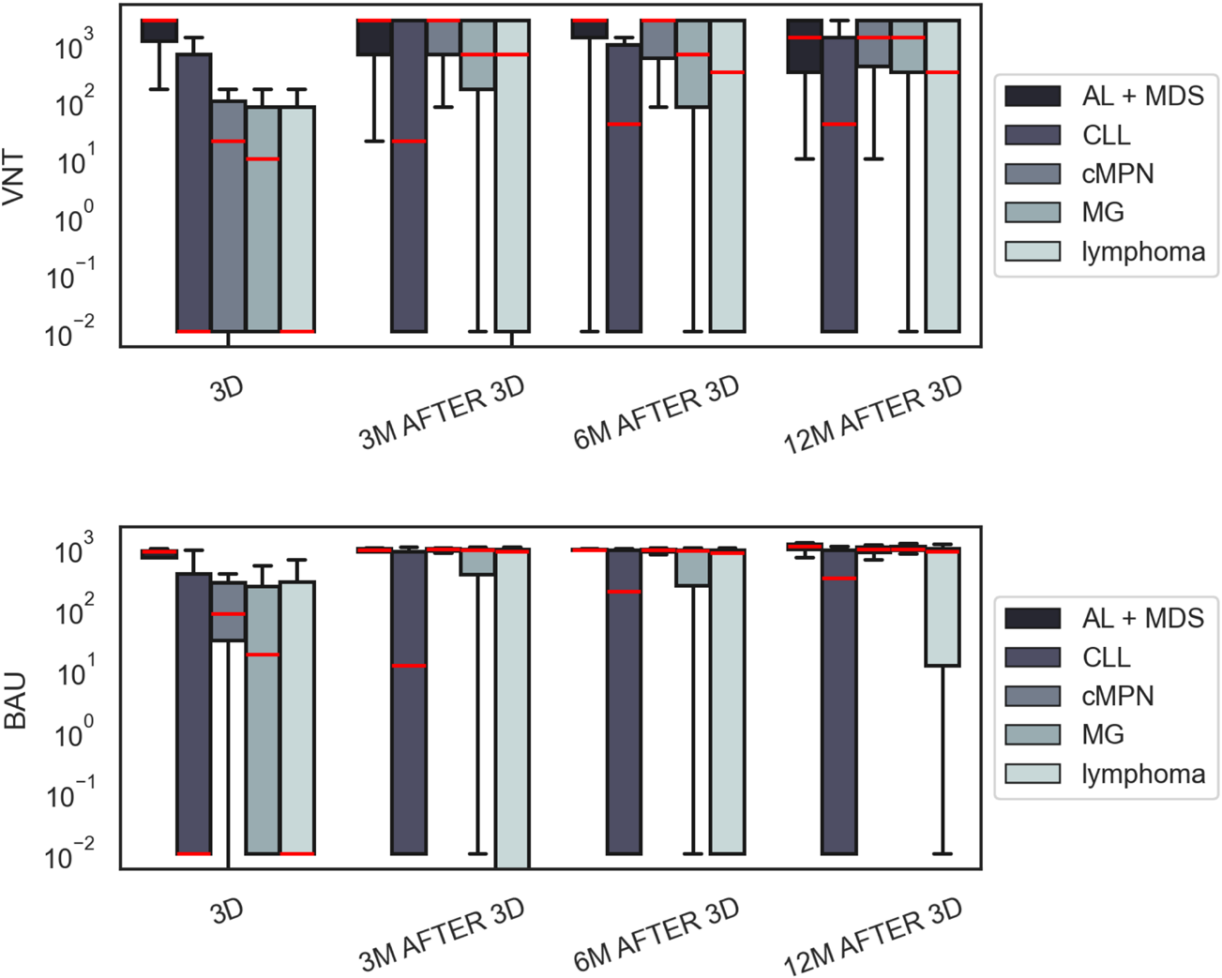
BAU and VNT levels for different patient subsets in the whole cohort. AL/MDS, acute leukemia/myelodysplastic syndrome; CLL, chronic lymphocytic leukemia; cMPN, chronic myeloproliferative neoplasms; MG, monoclonal gammopathies; VNT, Virus Neutralization Test.

Ondrej Šušol co‐authored the study design, wrote the article, supervised the vaccination program, collected patient data, and contributed to the statistical evaluation. Barbora Šušolová co‐authored the study design, supervised the vaccination program, collected patient data, and revised the final manuscript. Ondřej Klempíř performed the statistical evaluation and generated Figures 2, 3, 4 and revised the final manuscript. Roman Hájek co‐authored the study design, was the main study supervisor and revised the final manuscript. All other authors provided clinical supervision, participated on vaccination program and data collection and revised the final manuscript.

## Ethics Statement

The study has been approved by the local ethical committee (Ethical Committee of University Hospital Ostrava, Czech Republic) and all participants provided written informed consent prior to enrolment.

## Conflicts of Interest

Roman Hajek has had a consultant or advisory relationship with Janssen, Amgen, Celgene, AbbVie, BMS, Novartis, PharmaMar, and Takeda; has received honoraria from Janssen, Amgen, Celgene, BMS, PharmaMar, and Takeda; has received research funding from Janssen, Amgen, Celgene, BMS, Novartis, and Takeda. No other known COI from the other authors.

## Author Note

The number of authors exceeds six, because this work is a fruit of the cooperation of many members of our team. Given the nature of COVID‐19 pandemic, we have made maximal effort to vaccinate patients in the shortest possible time. Therefore, all contributing members are listed.

## Data Availability

The data that support the findings of this study are available from the corresponding author upon reasonable request.
